# Comparison between sensory and motor transcutaneous electrical nervous stimulation on electromyographic and kinesiographic activity of patients with temporomandibular disorder: a controlled clinical trial

**DOI:** 10.1186/1471-2474-14-168

**Published:** 2013-05-15

**Authors:** Annalisa Monaco, Fabrizio Sgolastra, Davide Pietropaoli, Mario Giannoni, Ruggero Cattaneo

**Affiliations:** 1 University of L’Aquila Department of Health Sciences, Via Vetoio 1, Italy, L’Aquila 67100, Italy

**Keywords:** Surface electromyography, Kinesiography, Temporomandibular disorder, Transcutaneous electrical nervous stimulation

## Abstract

**Background:**

The purpose of the present controlled clinical trial was to assess the effect of a single 60 min application of transcutaneous electrical nervous stimulation (TENS) at sensory stimulation threshold (STS), compared to the application of motor stimulation threshold (MTS) as well as to untreatment, on the surface electromyographic (sEMG) and kinesiographic activity of patients with tempormanbibular disorder (TMD).

**Methods:**

Sixty female subjects, selected according to the inclusion/exclusion criteria, suffering from unilateral TMD in remission were assigned to MTS, STS or untreatment. Pre- and post-treatment differences in the sEMG activity of temporalis anterior (TA), masseter (MM), digastric (DA) and sternocleidomastoid muscles (SCM), as well in the interocclusal distance (ID), within group were tested using the Wilcoxon test, while differences among groups were assessed by Kruskal-Wallis test; the level of significance was set at p ≤ 0.05.

**Results:**

Significant pre- and post-treatment differences were observed in MTS and STS groups, for TA and MM of both sides; no significant difference was detected between MTS and STS groups. Kinesiographic results showed that the vertical component of ID was significantly increased after TENS in MTS and STS groups.

**Conclusions:**

STS TENS could be effective, as well as MTS, in reduce the sEMG activity of masticatory muscles and to improve the ID of TMD patients in remission. Future studies are needed to confirm the results of the present study. Clinical relevance. The present study demonstrates that the application of TENS is effective in reduce the sEMG activity, as well as in increasing the ID of patients with TMD; our study did not support superior effectiveness of MTS or STS.

**Trial registration:**

ClinicalTrials.gov: NCT01832207

## Background

Temporomandibular disorder (TMD) is a collective term that embraces a number of clinical problems that involve the masticatory muscles, the temporomandibular joint and the associated structures. [1] The diagnosis of TMD is mainly based on clinical examination, even if additional auxiliary tools are available for a supplemental clinical investigation. Among those, surface electromyography (sEMG) has been proposed as supplemental tool in TMD diagnosis: despite the fact that the reliability of sEMG recordings from masticatory muscles is still lacking general consensus [2,3], since several issues, which are related to selectivity, reliability, and interpretation of sEMG signals, remain to be resolved [4,5], it has been suggested that sEMG could provide an objective recording of muscular activity [6-8], at rest and during functional activity, in a non-invasive way.

Transcutaneous electrical nervous stimulation (TENS) has been suggested as a treatment strategy in the therapy of TMD [9-11], since it has been showed to produce an antalgic effect in symptomatic patients and a positive relaxing effect on the masticatory muscles [12]. In clinical practice and research investigation, TENS has been administered at a variety of intensities as well of threshold of stimulation, both for antalgic [13,14] and relaxing purposes [15-17]. However, the effectiveness of TENS in reducing the sEMG activity of masticatory muscles, in patients with TMD, is still a debated question: differences, in terms of settings and types of TENS applications, among studies have been suggested to play an important role in explaining the contrasting findings, that have been observed in the literature [18-20]: interestingly, two controlled studies [15,17], that recruited patients with symptomatic and asymptomatic TMD, respectively, using the same treatment design, that consisted of a 60 min application of low intensity TENS with a motor threshold of stimulation (MTS), reported a significant reduction of sEMG activity of masticatory muscles; in contrast, another controlled study, that recruited patients with symptomatic TMD [16], using a 45 min application of high intensity TENS with a sensorial threshold of stimulation (STS), observed a significant reduction of pain intensity, and reported no significant differences with regard to the muscular activity in the group receiving TENS application.

Interestingly, Moran and coworkers [21] showed that TENS-mediated pain relief has a dose–response relationship, suggesting that intensity as well as threshold stimulation could influence the effectiveness of TENS application. These observations, could have important implications in clinical practice and research, since no optimal dosage as well as threshold of stimulation have been defined in the treatment of patients with TMD. Accordingly, no study is available to compare the effect of different threshold of stimulation on muscular activity of patients with TMD.

Previously [17], we showed that MTS is effective in reducing the sEMG activity of masticatory muscles; in the present study we assess, the effect of motor threshold of stimulation (MTS) TENS, compared to sensory threshold application (STS) TENS on the muscular activity of masticatory and neck muscles, of patients with TMD.

## Methods

### Subjects

This study was conducted in accordance with the Declaration of Helsinki. The Committee on Ethics in Science of the University of L’Aquila, L’Aquila, Italy approved the study and informed consent was obtained from each subject.

Sixty female subjects suffering from unilateral TMD in remission at least from 3 months, aged 24 to 30 years-old (median age = 26 years), were recruited and divided into three groups: twenty patients were assigned to a single session of 60 m of MTS TENS; twenty patients undergone a single session of 60 m of STS TENS; twenty patients received a delayed TENS treatment after the end of the study, and, therefore, this group received no treatment during the entire duration of the study and acted as control.

### Selection criteria

Considering that jaw elevator muscle activity can be influenced by oro-facial pain [22], gender [23], age [24], occlusion [25], and hemispheric-dominance [26], only patients that fulfilled the following inclusion criteria were included in the study: age less than 30 years; female gender; right-handed (7–10 points in Edinburgh inventory) [27]; presence of complete permanent dentition, with the exception of the third molars; normal occlusion; and diagnosis of unilateral arthrogenous TMD on the Research Diagnostic Criteria for TMD (RDC/TMD) [28,29], Axis I, groups II and III. Patients were excluded from the study if they met one or more of the following exclusion criteria: having pacemaker or other electrical devices, previous experience of TENS or biofeedback, systemic diseases, history of local or general trauma, neurological or psychiatric disorders, muscular diseases, cervical pain, bruxism, diagnosed by the presence of parafunctional facets and/or anamnesis of parafunctional tooth clenching and/or grinding; pregnancy, assumption of anti-inflammatory, analgesic, antidepressant or myorelaxant drugs, fixed or removable prostheses, fixed restorations that affected the occlusal surfaces, or previous or concurrent orthodontic or orthognathic treatment.

### sEMG, TENS and kinesiographic measurements

All examinations were performed by one examiner (A. M.), using an 8-channel surface electromyograph with simultaneous acquisition, common grounding to all channels, and filters of 50 Hz electromyography (K7/EMG, Myotronics-Noromed, Inc., Tukwila WA, USA), with disposable electrodes (Duotrode, bipolar surface electrodes Ag-AgCl, 20 mm center to center distance, Myotronics-Noromed, Inc., Tukwila WA, USA), for sEMG recording. The right masseter (RMM), left masseter (LMM), right anterior temporal (RTA), left anterior temporal (LTA), right digastric (RDA), left digastric (LDA), right sternocleidomastoid (RSCM), and left sternocleidomastoid (LSCM) muscles were recorded. The sEMG recordings and muscle activity was expressed as the root mean square (RMS) of the amplitude, expressed in μV [30]. Kinesiographic recordings were performed using a kinesiograph (K7/CMS; Myotronics-Noromed, Inc., Tukwila, WA, USA) that measures jaw movements with an accuracy of 0.1 mm. An array of lightweight (113 gr) with multiple sensors and containing 8 magnetic sensors, tracked the motion of a magnet (CMS Magnet; Myotronics-Noromed, Inc., Tukwila WA, USA), that was attached at the lower inter-incisor point. The kinesiograph was interfaced with a computer for data storage and subsequent software analysis (K7 Program, Myotronics-Noromed, Inc., Tukwila WA, USA).

### Positioning of sEMG, TENS electrodes and kinesiographic array

The electrodes determine, to a large extent, the quality of the recordings [31]. Electrodes were positioned on LMM, RMM, LTA and RTA, as described by Castroflorio et al. [6], as well on RDA, LDA [32], LSC and RSC [33,34], A template was used to enabled the exact reposition of the electrodes, in the occurrence of malfuctioning. The ground electrode, which was larger than the others and ensured a very good contact with the skin, was positioned on the subject’s forehead to ensure a common reference to the differential input of the amplifier. The kinesiographic array was mounted on the subject's head, and the optimal position of the magnet for the recording of kinematic movements was monitored by software.

Due to the noise inherent with the sEMG recording, a special attention was paid to obtain reproducible and standardized recordings; approximately 15% of the electrodes required a relocation after new degreased, dry, jelly, and electrode fixation; however, to obtain a reliable sEMG recording, the reliability of signal captation of each electrodes was tested by a noise test software (K7 Program, Myotronics-Noromed, Inc., Tukwila WA, USA): only when the software gave the absence of noise (corresponding to the value provided by the software of 0.0), the sEMG recording was started.

### Stimulation procedure

For TENS application a J5 Myomonitor TENS Unit device (Myotronics-Noromed, Inc., Tukwila, WA, USA), with disposable electrodes (Myotrode SG Electrodes, Myotronics-Noromed, Inc., Tukwila, WA, USA) was used: this low-frequency neurostimulator generates a repetitive synchronous and bilateral stimulus, delivered at 1.5 s intervals, with a variable amplitude of approximately 0–24 mA, a duration of 500 μs and a frequency of 0.66 Hz. The two electrodes for TENS were placed bilaterally over the cutaneous projection of the notch of the V pair of cranial nerves, that is located between the coronoid and condylar process and was retrieved by manual palpation of the zone anterior to the tragus; a third grounding electrode was placed in the center of the back of the neck [15]; however, since in the area of application of TENS, fibers of VII pair of cranial nerves are present, MTS TENS resulted in the motor stimulation of jaw elevator and facial muscles.

The stimulation procedure was performed under kinesiographic recording, in order to assess the achievement and the absence of motor stimulation in the MTS and STS group, respectively. In both MTS and STS groups the amplitude of TENS stimulation was reached starting from 0, with the stimulator turned on and the rheostat, which controls amplitude, positioned on 0; thus, the amplitude of stimulation was progressively increased of 0.6 mA/s: in the MTS group the stimulation was progressively increased until the contraction of the elevator muscles of the jaw was observed on the kinesiographic track.

In the STS group, the stimulation was progressively increased until the patients reported the sensation of pricking: a particular attention was paid to avoid the reaching of the threshold of motor stimulation: indeed, in this group, if any movement of the investigated muscles was observed during the kinesiographic recording, the patients was excluded from the study.

### Recording procedure

Electromyographic and kinesiographic recordings were conducted with patients comfortably seated on a wooden chair with a straight back; patients were asked to assume a relaxed position of the head. The measurements were performed in a silent and comfortable environment. For sEMG recording, three consecutive tracks, with a duration of 15 s, and withouth any interposing phasic event, such as swallowing, voluntary movement or clenching, were acquired with the mandible of the patient in rest position. During the recordings, each subject was asked to keep his/her eyes closed, maintaining a slight contact between his/her lips. The participants received these instructions before the recordings. For each patient, sEMG and kinesiographic measurements were recorded before and immediately after the application of TENS. Patients in the control group received no treatment, and underwent sEMG and kinesiographic recording at baseline and immediately after TENS.

**Figure 1:**
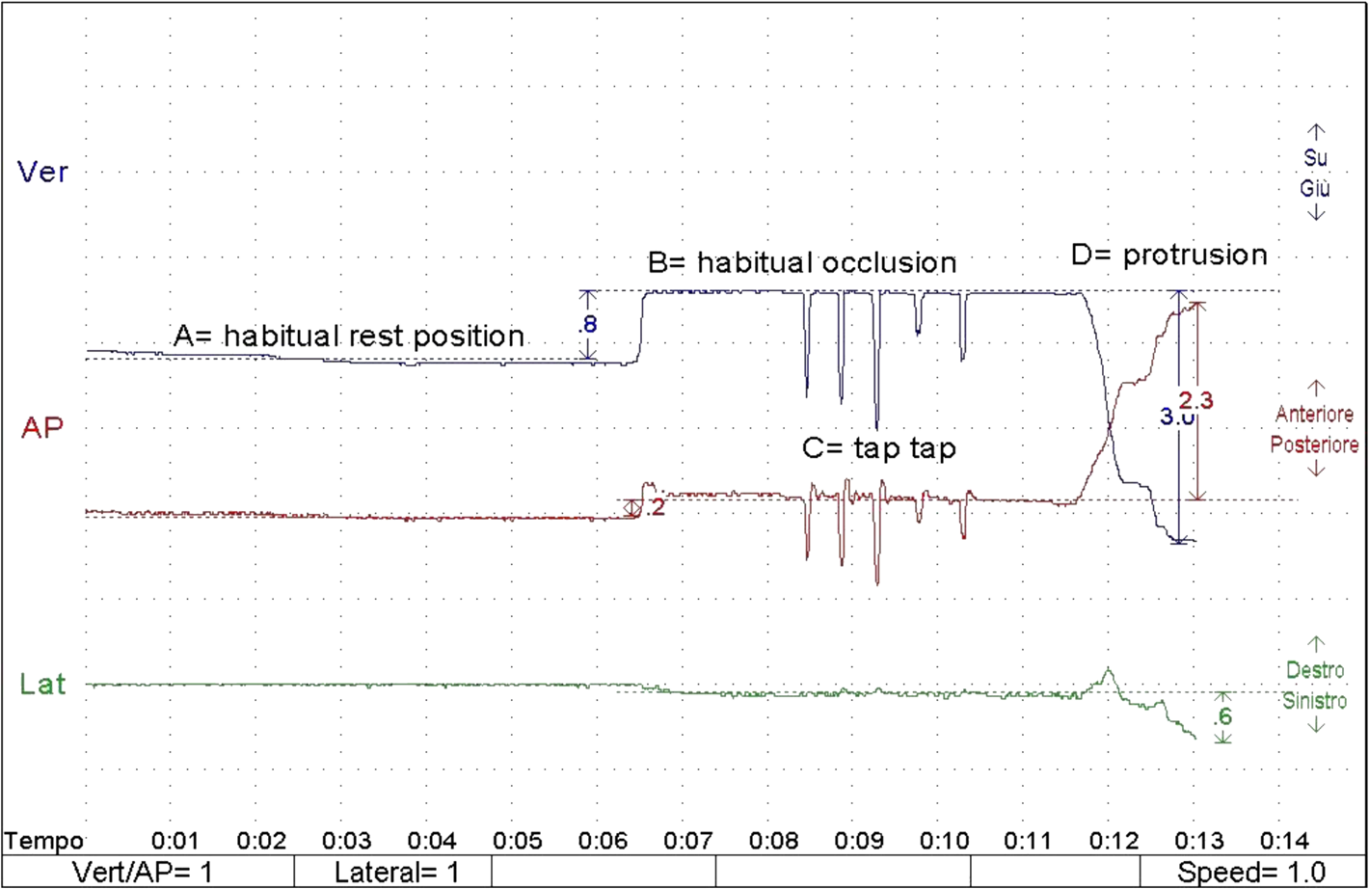
**Kinesiographic track.** Ver (Verticality/Blu line) refer to vertical component of the interocclusal distance; AP (Anterior-Posteriority/Red line) refer to anterio-posterior component of interocclusal distance. Lat (Laterality/Green line) refer to lateral component of interocclusal distance. Line at the beginnig of the track refer to basal position of the mandible at rest position.

For kinesiographic recording, the patients were asked to close their eyes and, starting from the mandible rest position, to occlude teeth; after 2 s from occlusal contact, the patients were asked to quickly open and return in occlusal contact (tap-tap phase); for each patient, at least 3 consecutive tap-tap phases were recorded; the achievement of occlusal contact during each tap-tap phase was defined as the presence on the vertical component (Figure 1, Blu line) of the kinesiographic tracing of an horizontal line located at the same height of the first occlusal contact (Figure 1). After the tap-tap phase, the patients keep their habitual occlusal position for at least 1 s, then, they were asked to protrude the mandible. Kinesiographic recording was started at the end of the TENS session.

For kinesiographic measurements, the interocclusal distance (ID) was recorded in its threedimensional component: verticality (defined as the difference between basal and the highest level of the blue line) (Figure 1), anterior-posteriority (defined as the difference between basal and highest level of the red line) (Figure 1), and verticality/anterior-posteriority (V/AP) ratio.

### Study design

**Figure 2:**
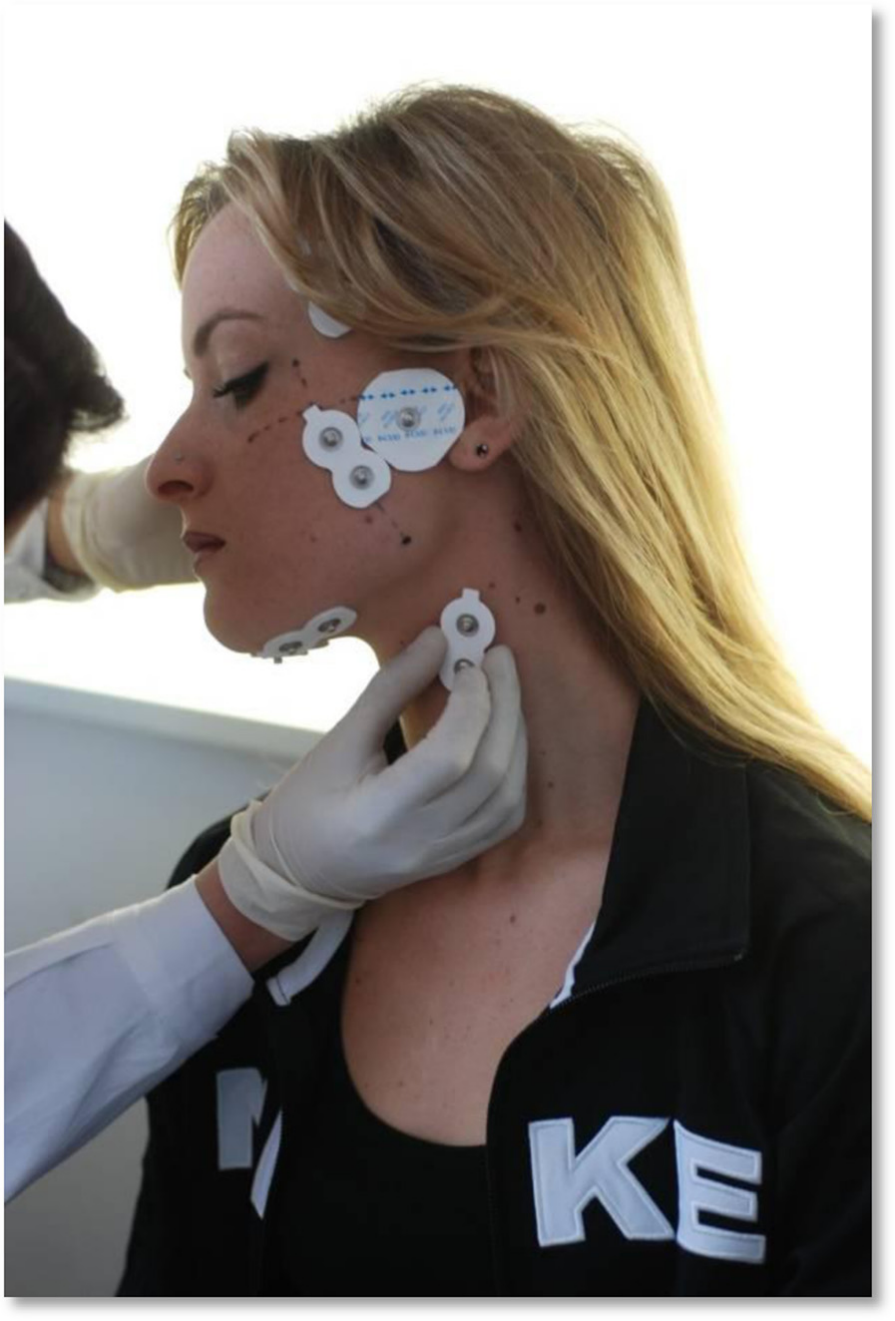
sEMG and TENS electrodes positioning.

**Figure 3:**
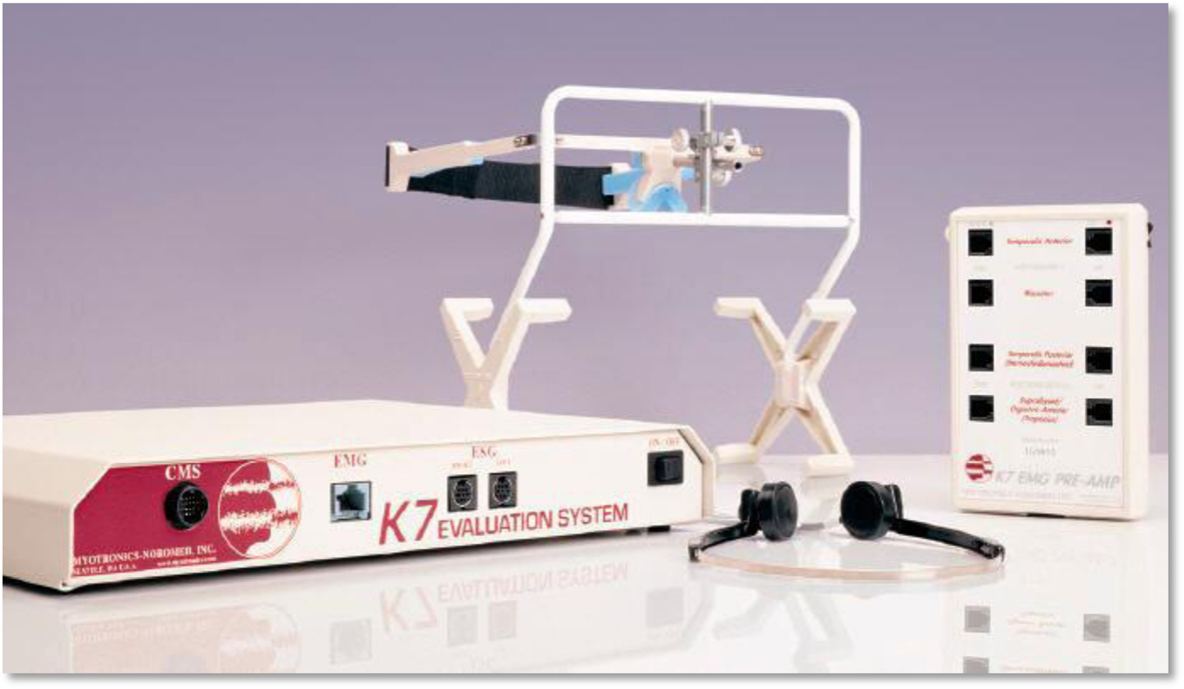
K7 electromyographic and kinesiographic instruments.

TENS electrodes were placed in all patients, but the stimulation was performed only in the MTS AND STS groups. The stimulation procedure was performed by the same operator who placed the electrodes (Figures 2 and 3).

### Sample size calculation

The sample size calculation determined that 16 subjects per treatment arm would provide a 80% power to detect a true difference of 0.6 μV among the three study groups using the reduction in the mean RMS between LTA and RTA as the primary outcome variable, assuming that the common standard deviation is 0.6 μV. Accordingly, a sample of 20 subjects per arm was recruited to overcome the possibility of dropouts.

### Statistical analysis

Statistical analysis was performed using STATA 10 (StataCorp LP, College Station, TX, USA). The level of significance was assumed to be p ≤ 0.05 for all tests.The Shapiro-Wilk test indicated a non normal distribution of the sEMG and kinesiographic data, as well of the patient’s age in the three groups; therefore, pre- and post-treatment within group differences in the sEMG and kinesiographic data were analyzed using the Wilcoxon test. Differences in the sEMG, kinesiographic data and age among the three groups, were assessed by Kruskal-Wallis test. Kinesiographic and sEMG data are expressed as means and standard deviations (SD). Intraclass coefficient correlation (ICC) was calculated to estimate the intra- and inter-sessional reproducibility, according to the protocol suggested by Castroflorio and coworkers [35].

## Results

All sixty female patients completed the study and no droput occurred. The mean age was 25.5±1.3 in MST group, 26.3 ± 1.2 in STS group and 25.4 ± 1.1 in the control group; no significant differences were observed in age among the three groups (p > 0.01).

### Reproducibility

At the first sEMG recording, the mean ICC for investigated muscles was 0.832, while in the second recording the mean ICC was 0.803; globally, the ICC was 0.814.

### SEMG and kinesiographic findings

At baseline, no significant difference in sEMG and kinesiographic measurements was observed among the three groups. In the MTS group, motor stimulation was reached in 15–20 s, while in the STS the group sensory stimulation was reached in 5–10 s.

Immediately after TENS, a reduction in RMS was observed for all investigated muscles in MTS and STS groups; in the control group, only RMM, LDA and RDA showed a reduction in RMS values, even if these changes were not statistically significant. Significant pre – post-treatment differences were observed in the MTS and STS groups, for LTA RTA LMM and RMM. Kruskal-Wallis test revealed that RMS values of LTA RTA LMM and RMM in the MTS and STS groups were significantly reduced, in comparison with control group; however, no significant differences were observed between MTS and STS groups at baseline or at post-treatment timepoint (Table 1).

**Table 1:** Values of sEMG activity in the MTS, STS and control group

	**MTS group**		**STS group**		**CONTROL group**	
	**Baseline**	**After TENS**	**Baseline**	**After TENS**	**Baseline**	**After TENS**
LTA	2.79 (1.69)	1.62 (1.08) ^a,b^	2.91 (1.48)	1.70 (0.99) ^a,b^	2.69 (1.25)	3.18 (1.93)
RTA	2.87 (1.76)	1.71 (1.13) ^a,b^	2.83 (1.46)	1.64 (1.07) ^a,b^	2.93 (1.57)	3.36 (1.67)
LMM	1.59 (0.91)	1.17 (0.64) ^a,b^	1.59 (1.17)	1.12 (0.78) ^a,b^	1.37 (1.19)	1.39 (1.26)
RMM	1.47 (1.01)	1.11 (0.77) ^a,b^	1.50 (1.19)	1.14 (0.54) ^a,b^	1.51 (1.19)	1.42 (1.33)
LSM	2.44 (1.81)	2.23 (2.40)	2.51 (1.71)	2.52 (2.05)	2.19 (1.30)	2.38 (1.97)
RSM	2.28 (1.74)	2.24 (2.74)	2.39 (2.33)	2.27 (2.26)	2.46 (1.92)	2.65 (1.81)
LDA	1.99 (1.17)	1.79 (1.11)	1.91 (1.12)	1.82 (0.75)	1.79 (0.76)	1.64 (0.68)
RDA	2.16 (1.26)	1.95 (1.25)	1.98 (1.21)	1.76 (089)	1.94 (1.35)	1.89 (1.09)

**Legend:** MTS, motor threshold of stimulation; STS, sensorial threshold of stimulation; LTA, left temporalis anterior; RTA, right temporalis anterior; LMM, left masseter muscle; RMM, right masseter muscle; LSM, left sternocleidomastoi muscle; RSM, right sternocleidomastoi muscle; LDA, left digastric anterior; RDA, right digastric anterior.

Letters refer to significant change pre-post treatment (a) or among groups (b).

Kinesiographic results showed that the vertical component of ID, as well as V/AP ratio were significantly increased after TENS, in both MTS and STS groups; significant differences were found for MTS and STS groups in comparison with control group (Table 2); no significant differences for any kinesiographic parameter was observed between MTS and STS groups.

**Table 2:** Kinesiographic values in the MT TENS, ST TENS and control group

	**MTS TENS**		**STS TENS**		**CONTROL**	
	**Baseline**	**After TENS**	**Baseline**	**After TENS**	**Baseline**	**After TENS**
Verticality	1.23 (0.72)	3.03 (1.17)^a,b^	1.25 (0.74)	2.94 (1.14) ^a,b^	1.25 (0.79)	1.27 (1.09)
Anterior-posteriority	0.61 (0.36)	0.78 (0.51)	0.58 (0.47)	0.70 (0.38)	0.59(0.44)	0.64 (0.39)
V/AP Ratio	2.36 (1.18)	4.42 (3.06) ^a,b^	2.15 (1.23)	4.20 (2.97) ^a,b^	2.24 (1.18)	2.09 (1.46)

**Legend:** MTS, motor threshold of stimulation; STS, sensorial threshold of stimulation; V/AP, verticality - anterior-posteriority ratio.

Letters refer to significant change pre-post treatment (a) or between test (MST or SST) and control group (b).

## Discussion

In the present study, the effect of two different types of TENS stimulation on the sEMG activity as well on the kinesiographic pattern of patients with TMD in remission was investigated; the results suggest that both MTS and STS are effective in reducing the activity at rest of LMM, RMM, LTA and LTA, as well in increasing the inter-occlusal distance; furthermore, no significant difference was found between MTS and STS. These findings are in agreement with those achieved by other studies [15,16,35,36]; in particular Cooper and Kleinberg [15] found that MTS application reduced the sEMG activity of masticatory muscles, as well as symptoms; however, even if the effect of MTS on sEMG values were evaluated before and immediately after the MTS application, changes in symptoms were evaluated after one and three months, during which patients underwent an additional treatment with orthosis; therefore, it is difficult to define the amount of reduction in sEMG activity as well in symptoms, that could be attributed to MTS application. Rodrigues et al. [16] evaluated the effects of TENS on sEMG activity and pain of 19 patients suffering from TMD: a significant reduction on pain level, as well on the sEMG activity of LTA and RTA, at rest, was observed; however, this study used a high intensity STS with a time of application of 45 min.; therefore, the discrepancies in the settings of TENS make impossible comparisons with our findings.

Some concerns have been claimed on the validity of the sEMG recording in the diagnosis and monitoring of TMD [2,3,37]; these concercens are mainly related to the reliability of the sEMG recording and to its reproducibility: in the present study, the reproducibility of the sEMG measurements was assessed through the protocol suggested by Castroflorio et al. [38]; accordingly, a mean ICC of 0.814 was obtained; this value is in agreement to that reported by Castroflorio et al.[38] and could be considered as indicative of an excellent reproducibility [39].

With regard to the condition of the sEMG recording, no general consensus has been reached in the literature on what condition should be the most appropriate and reliable for the reproducibility of the sEMG recording: even if clenching has been suggested to be a more reliable and standardized condition for sEMG recording, than rest position [7], it has been observed that in situations of chronic muscular pain, muscular contraction ability is reduced due to the decrease in activity of the agonist muscles and the increase in activity of the antagonist muscles [40-42]; in the present study we selected only asymptomatic patients, that were in remission from chronic pain accordingly and did not use clenching as the condition for sEMG recording, but rest position of the mandible, that has been suggested to be more reliable in asymptomatic patients [43].

To enhance the internal validity of the study and to obtain an homogeneous sample, we used strict and rigorous inclusion/exclusion criteria in the selection of patients, and performed a sample size calculation, which indicated that a minimum of 16 patients per group would be required; it has been reported that TMDs occur more frequently in women than man [44]: this potential confounding factor, as well as other like age, ethnicity, gender, hemispheric-dominance, missing teeth, occlusal alteration, the presence of systemic condition that could affect the activity of muscles, nervous system and joints, has been excluded to homogenize all groups under study. These strict inclusion criteria were required considering that the present was a pilot study.

The present study has an important limitation, since it was not a randomized double-blind clinical trial: the randomization process is performed to assign participants to study groups, such that the groups are balanced for known and unknown risk factor, to minimize bias; the absence of randomization may have introduced a bias into the study.

Based on the findings of the present study, the application of a single session of 60 min of STS is as effective as MTS, in reducing the sEMG activity of LTA, RTA, LMM and RMM at rest and in increasing the ID in patients with TMD in remission. However, further studies are required to assess the effect of STS, compared to MTS, on the sEMG and kinesiographic pattern of patients with symptomatic TMD.

## Conclusions

STS TENS could be effective, as well as MTS, in reduce the sEMG activity of masticatory muscles and to improve the ID of TMD patients in remission. Future studies are needed to confirm the results of the present study. Clinical relevance. The present study demonstrates that the application of TENS is effective in reduce the sEMG activity, as well as in increasing the ID of patients with TMD; our study did not support superior effectiveness of MTS or STS.

## Abbreviations

TMD: Temporomandibular disorder; sEMG: Surface electromyography; TENS: Transcutaneous electrical nervous stimulation; MTS: Motorial threshold of stimulation; STS: Sensorial threshold of stimulation; V/AP: Verticality/antero-posteriority; ID: Interocclusal distance; LTA: Left temporalis anterioris; RTA: Right temporalis anterioris; LMM: Left masseter muscle; RMM: Right masseter muscle; RSCM: Right sternocleidomastoid; LSCM: Lfet sternocleidomastoid; RDA: Right digastric; LDA: Left digastric.

## Competing interest

The authors declare that they have no competing interests.

## Author's contributions

GM conceived the study. AM and FS collected the data. DP performed the surface electromyographic evaluation and RC performed the statistical analysis. All the authors concepted the manuscript and were involved in writing the paper. All authors read and approved the final manuscript.

## Pre-publication history

The pre-publication history for this paper can be accessed here:

http://www.biomedcentral.com/1471-2474/14/168/prepub
